# Achieving Biocompatible SABRE: An in vitro Cytotoxicity Study

**DOI:** 10.1002/cmdc.201700725

**Published:** 2018-01-18

**Authors:** Anand Manoharan, Peter J. Rayner, Wissam Iali, Michael J. Burns, V. Hugh Perry, Simon B. Duckett

**Affiliations:** ^1^ Department of Chemistry University of York Heslington York YO10 5DD UK; ^2^ School of Biological Sciences University of Southampton Southampton UK

**Keywords:** biocompatibility, biphasic catalysis, cytotoxicity, hyperpolarization, SABRE

## Abstract

Production of a biocompatible hyperpolarized bolus for signal amplification by reversible exchange (SABRE) could open the door to simple clinical diagnosis via magnetic resonance imaging. Essential to successful progression to preclinical/clinical applications is the determination of the toxicology profile of the SABRE reaction mixture. Herein, we exemplify the cytotoxicity of the SABRE approach using in vitro cell assays. We conclude that the main cause of the observed toxicity is due to the SABRE catalyst. We therefore illustrate two catalyst removal methods: one involving deactivation and ion‐exchange chromatography, and the second using biphasic catalysis. These routes produce a bolus suitable for future in vivo study.

## Introduction

Clinical magnetic resonance imaging (MRI) is at the forefront of disease diagnosis. It uses strong magnetic fields, radio waves, and field gradients to give detailed images of human anatomy. However, due to the inherent low sensitivity of MRI, almost all clinical applications detect water due to its high concentration in the body. Hyperpolarization methods turn typically weak MRI responses into strong signals and thus open the door to new diagnostic techniques. For example, dynamic nuclear polarization (DNP) has been used to create the signal strength necessary to track the in vivo metabolism of pyruvate in prostate tumors,^1^ whilst spin exchange optical pumping (SEOP) of noble gases has allowed the diagnosis of pulmonary diseases.^2^

**Figure 1:**
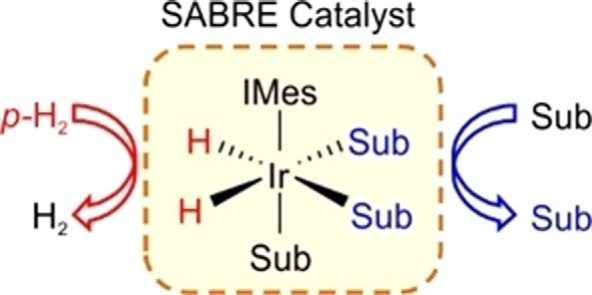
Schematic representation of the SABRE effect.

An alternative low‐cost approach to hyperpolarization uses parahydrogen (*p*‐H_2_) to create a non‐Boltzmann nuclear spin distribution without changing the identity of the molecule of interest. This technique is known as signal amplification by reversible exchange (SABRE) and is shown schematically in Figure 1.^3^ It can enhance the signals detected by MRI and nuclear magnetic resonance (NMR) spectroscopy across a wide range of nuclei such as ^1^H, ^13^C, ^15^N and others.^4^ It works by harnessing the latent polarization of *p*‐H_2_ through binding to a metal catalyst, typically [Ir(H)_2_(Sub)_3_(IMes)]Cl,^5^ as hydride ligands. Simultaneous binding of the substrate allows spontaneous transfer of polarization through the scalar coupling network at low magnetic fields.^6^ Subsequent substrate dissociation from the catalytic complex allows buildup of hyperpolarized substrate in solution.

A recent study showed that ^2^H labeling a series of nicotinamide and methyl nicotinate molecules can simultaneously improve their SABRE‐enhanced NMR and MRI detection and decrease magnetic relaxation. The optimal substrate in this study was methyl‐4,6‐*d*_2_‐nicotinate (***d***_**2**_ **‐MN**), which gave up to 50 % polarization in conjunction with *T*_1_ relaxation values approaching 2 minutes.^7^ This makes ***d***_**2**_ **‐MN** an ideal candidate for in vivo detection. However, despite some applications of methyl nicotinate (**MN**) in cosmetic and veterinary pharmaceutics, the effect of selective deuteration has not been studied.

The isotopic labeling of drug molecules is a well‐established route to modify their safety and/or efficacy.^8^ Deuteration primarily affects the biological fate of drugs that are metabolized through a pathway that involves hydrogen–carbon bond breaking, as significant rate changes can occur due to the kinetic isotope effect. The metabolic pathway of nicotinic acid derivatives typically proceeds via formation of the *N*‐oxide, though routes involving 6‐hydroxy species are also known. For this reason the toxicity of ***d***_**2**_ **‐MN** in comparison with **MN** is determined here using in vitro cytotoxicity analysis.

Another potential source of toxicity that arises from the SABRE technique is the iridium catalyst itself. Whilst some data on the adverse effects of iridium salts has been reported^9^, little information is known about organo‐iridium complexes.^10^ Therefore, we present a thorough investigation into the potential toxicity of the metal catalyst in conjunction with solvent and substrate effects. We determine the biocompatibility of the SABRE reaction by performing in vitro cytotoxicity analysis on human cell lines and present a method for depletion of the catalyst from solution in order to create a biocompatible bolus that could be progressed to in vivo measurement.

## Results and Discussion

### Evaluating the cytotoxicity of SABRE substrate

Methyl‐4,6‐*d*_2_‐nicotinate (***d***_**2**_ **‐MN**) has been reported to give the highest ^1^H polarization levels by SABRE to date.^7^ From a biological perspective, **MN** is widely used as rubefacient in cosmetics due to its percutaneous penetration properties upon topical application and moreover known for its vasodilatory effects at lower doses and inflammatory response at higher doses.^11^ Given the substantial SABRE enhancement levels and biological applicability, ***d***_**2**_ **‐MN** is considered an ideal candidate for probing on in vitro/in vivo tests for SABRE detection. However, whether ***d***_**2**_ **‐MN** retains the characteristics of **MN** is still unclear. In this study, we use ***d***_**2**_ **‐MN** as a model substrate to ascertain the broader SABRE toxicology profile.

To establish the effect of selective deuteration on toxicity we began by determining the IC_50_ (half maximal inhibitory concentration) values of **MN** and ***d***_**2**_ **‐MN**. For this, well‐established cancer cell lines of human origin were treated with either **MN** or ***d***_**2**_ **‐MN** at varying concentrations for up to 48 h, and the viability was assessed by the MTT method.^12^ The results obtained for each cell line are shown in Table 1. The IC_50_ value for **MN** was found to range between 12.6–33.3 mm across the cell lines, and interestingly, despite the deuterium labeling, a comparison of the IC_50_ values indicates that the toxicity levels of ***d***_**2**_ **‐MN** are similar to those of **MN**. Importantly, both substrates have millimolar IC_50_ values (Table 1) and are within the concentration range used in a typical SABRE reaction (mm). It is noted here that these results are based on the solubility of the substrates directly in the high volume of the cell culture medium, and it is still unclear how the effect would be under the conditions of SABRE, in which different solvents are used. Nevertheless, these data provide a reference IC_50_ value for optimizing SABRE for further analysis. From this analysis, we conclude that deuteration of the methyl nicotinate has no quantifiable effect in modulating toxicity across the cell lines studied here.

**Table 1:** Comparing the IC_50_ values of methyl nicotinate (**MN**) vs. methyl‐4,6‐*d*_2_‐nicotinate (***d***_**2**_ **‐MN**) in human cancer cell lines.

Cell Line	IC_50_ [mm]	
	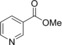	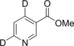
	**MN**	***d***_**2**_ **‐MN**
A549	13.6	17.4
MCF7	33.3	25.2
HeLa	21.9	14.9
MDA‐MB‐231	12.6	12.3

### Effect of SABRE solvents on cell viability

SABRE‐induced polarization levels are typically highest in alcohol solvents such as [D_4_]methanol or [D_6_]ethanol.^7^, ^13^ However, some applications can produce hyperpolarized substrates in aqueous solution, though enhancement levels are typically reduced.^14^ To create a biocompatible hyperpolarized bolus with high polarization levels, it has been suggested that the hyperpolarization step should be carried out in [D_6_]ethanol prior to dilution with D_2_O.

**Figure 2:**
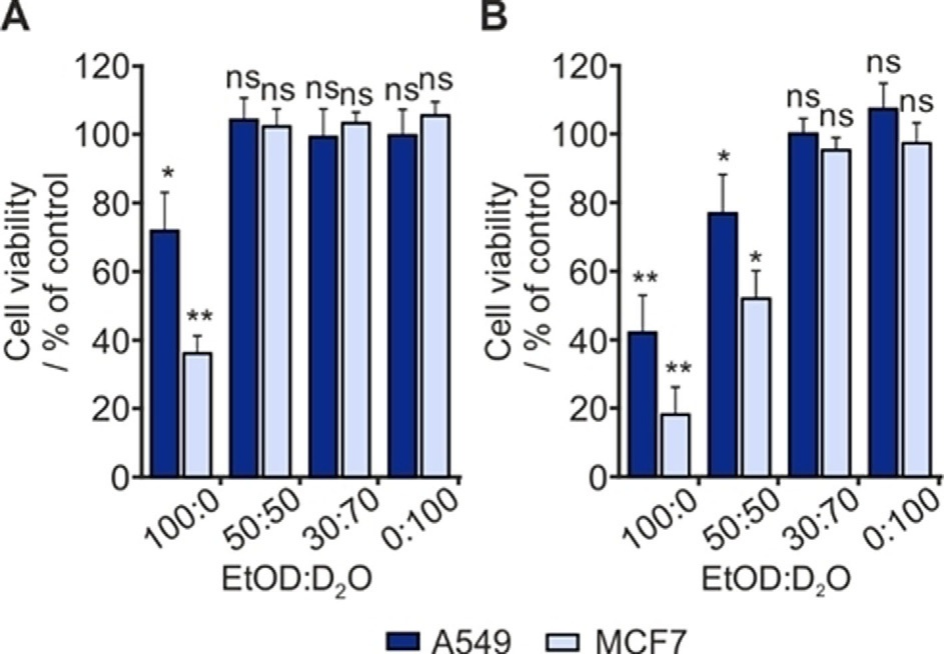
Effect of deuterated solvent mixture on cell viability: MTT cell viability assays performed on the indicated cell lines after A) 6 h and B) 24 h of treatment with deuterated solvents at various ratios. The final solvent volume in the cell culture medium was 10 %. EtOD=[D_6_]ethanol. Data are the mean+SD. *P<0.05, **P<0.005, ns: not significant vs. untreated control (100 % viable); one‐way ANOVA.

To assess the toxicity of these solvent mixtures, we performed an appropriate cell viability assay on A549 and MCF7 cells, which were treated with various dilutions of [D_6_]ethanol in D_2_O. As shown in Figure 2, the viability of both cell lines was significantly decreased if [D_6_]ethanol (100 %) was added to cell culture medium and treated for a short time (6 h). Conversely, over the same time period (6 h), 50 % [D_6_]ethanol in D_2_O (1:1) showed no change in cell viability. Extending the treatment durations to 24 h, however, significantly decreased the viability (Figure 2 B). We found that treatment of cells in a 30 % [D_6_]ethanol in D_2_O (30:70) solution did not show toxicity to cells over long treatment times relative to other deuterated solvent mixtures (Figure 2 B and Supporting Information Figure S1). Together the results indicate that a significant decrease in cell viability is evident when the solvent contained [D_6_]ethanol concentrations higher than or equal to 50 %. The behavior of analogous protio solvent mixtures is similar (Supporting Information Figure S2). From these in vitro cytotoxicity analyses on SABRE solvents, we conclude that to achieve optimal biocompatibility it is important to consider the duration of exposure on cells in culture (or in vivo) when performing SABRE using solvent with higher (>30 %) [D_6_]ethanol content.

As the optimal solvent mixture for biocompatibility determined here is 30 % [D_6_]ethanol in D_2_O, we wished to quantify the effects of using this solvent directly for the SABRE polarization of ***d***_**2**_ **‐MN**. Thus, we prepared a sample containing 5 mm [IrCl(COD)(IMes)], 20 mm ***d***_**2**_ **‐MN** in [D_6_]ethanol in D_2_O (30:70). After exposure to *p*‐H_2_ at 3 bar and 298 K in a magnetic field of 60 G we observed a 105±22‐fold total signal enhancement at 9.4 T. The corresponding ^1^H NMR spectra are shown in the Supporting Information (Figure S3). This is a significant decrease in polarization relative to the use of 100 % [D_6_]ethanol as solvent, and polarization under 3 bar *p*‐H_2_ gave a signal enhancement of >2800‐fold.^7^ As we have previously shown, further optimization may be achieved by using a higher pressure of *p*‐H_2_ and isotopically labeled catalysts, and work is ongoing to improve the polarization levels.

### Evaluating the cytotoxicity of substrate in solvent

**Figure 3:**
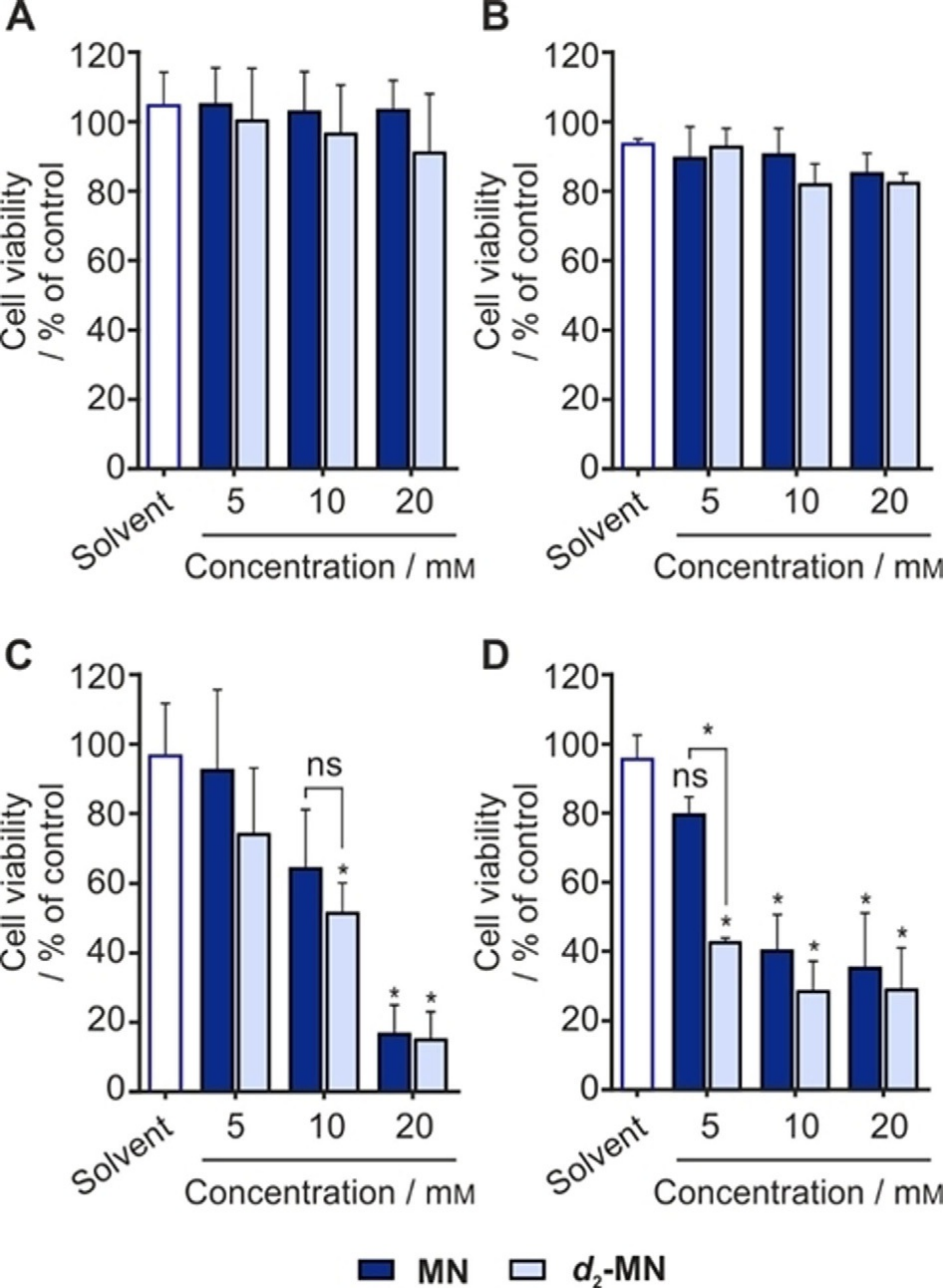
Cytotoxicity of alcohol‐solubilized methyl nicotinate: MTT cell viability data showing A), C) A549 and B), D) MCF7 cells treated for 6 h (upper panel) and 24 h (lower panel) with various dilutions of **MN** and ***d***_**2**_ **‐MN** solubilized in 30 % [D_6_]ethanol in D_2_O. The final solvent volume in the cell culture medium was 10 %. Data are the mean+SD from three independent experiments (*n*=3). Statistically significant differences from untreated control group (or from protio form of **MN**) are shown. *P<0.05, ns: not significant vs. untreated control group; one‐way ANOVA.

Having found that 30 % [D_6_]ethanol in D_2_O does not induce toxicity to cells in vitro, we then performed viability assays for ***d***_**2**_ **‐MN** dissolved in this solvent mixture. Again, we compared ***d***_**2**_ **‐MN** with **MN** to exclude any toxic effects that arise from the selective deuteration in ***d***_**2**_ **‐MN** when dissolved in alcohol solvents. Importantly, ***d***_**2**_ **‐MN** shows good solubility in this solvent composition and it did not decrease the viability of A549 and MCF7 cells when treated for up to 6 h (Figure 3 A,B, respectively). However, longer treatment times at concentrations higher than 5 mm are shown to affect the viability of both cell lines (Figure 3 C,D). Surprisingly, as shown in Figure 3 D, when compared with **MN**, ***d***_**2**_ **‐MN** induced a significant decrease in the viability of MCF7 cells at this concentration (5 mm) when treated for long time (24 h). This further indicates that deuteration might affect the toxicity effects of the substrate either by itself, or that toxicity is more pronounced as an additive effect when mixed in alcohol solvent at this long exposure time (24 h). Together, our data suggest that the in vitro cytotoxicity of ***d***_**2**_ **‐MN** in an [D_6_]ethanol/D_2_O solvent mixture depends on the duration of exposure on cells in culture. It is worth mentioning that the aim of our cytotoxicity assessment is to allow us to understand the transient effect these compounds or solvents play under the stated conditions. We are aware that in an in vivo setting, biocompatibility would be dependent on physiological status and the pharmacokinetics of the organism and the mechanism of action of the treated material. Nonetheless, the SABRE approach requires the contrast agent to stay in the body for a comparatively short time prior to excretion, as relaxation limits utility and further reduces toxicity concerns.

### Evaluating the biocompatibility of SABRE reaction mixture

**Figure 4:**
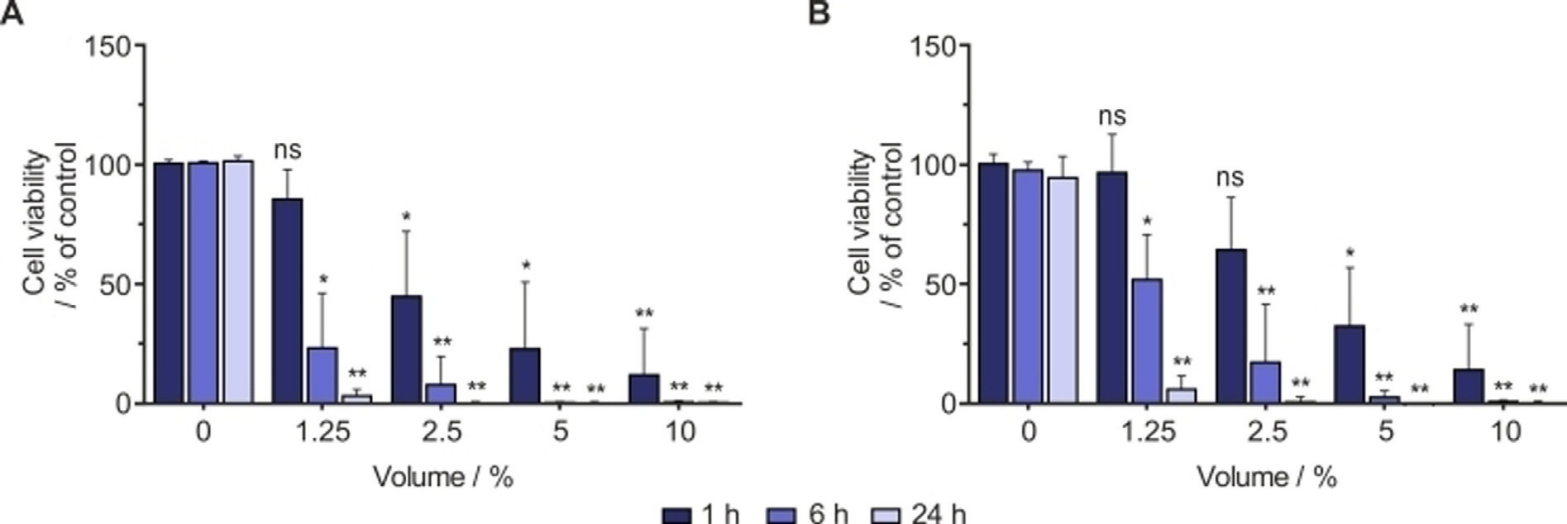
Evaluating the biocompatibility of SABRE reaction mixture: A) A549 and B) MCF7 cells were treated with various bolus volumes (0, 1.25, 2.5, 5, and 10 %) of SABRE reaction mixture, and cell viability was assessed 1, 6, and 24 h thereafter by MTT assay. Data are the mean+SD from three independent experiments (*n*=3). *P<0.05, **P<0.005, ns: not significant vs. untreated control (100 % viable); one‐way ANOVA.

Given that the cytotoxic dosage of ***d***_**2**_ **‐MN** in the solvent [D_6_]ethanol/D_2_O (30:70) is well above the amounts used for a typical SABRE reaction (considering only less than or equal to 10 % will be taken as a bolus for treatment) we sought to investigate the effect of the SABRE reaction mixture on A549 and MCF7 cell lines. For this, we prepared a typical SABRE solution containing 5 mm of [IrCl(COD)(IMes)] together with 20 mm ***d***_**2**_ **‐MN** in [D_6_]ethanol/D_2_O (30:70) and activated it with 3 bar H_2_. We exposed the cells to various bolus volumes (1.25, 2.5, 5, and 10 %) of the activated mixture and assessed the viability of the cells at different time periods by MTT assay. As illustrated in Figure 4, treatment with the SABRE reaction mixture over a short period of time (1 h) did not decrease the viability of A549 and MCF7 cells when the lowest volume (e.g., 1.25 %) was added to the cell culture medium. However, cells that were treated with 10 % bolus of the SABRE reaction mixture showed less viability at the same time point.

To distinguish the cytotoxic effect of the substrate and catalyst in the mixture we prepared, in parallel, analogous solutions containing various concentrations of either ***d***_**2**_ **‐MN** or [IrCl(COD)(IMes)] alone. Unfortunately, [IrCl(COD)(IMes)] is less soluble in [D_6_]ethanol/D_2_O (30:70) and, moreover, cannot form an active catalyst without the presence of a substrate when activated with H_2_. Under these conditions, a precipitate formed (Supporting Information Figure S4), and cytotoxicity assessment using this emulsion to treat cells is not expected to provide comparable results. Nevertheless, we treated the cells with a solution of ***d***_**2**_ **‐MN** alone, and no adverse effect was observed even at treatments over 24 h on both cell lines. This indicates that the deleterious effect of the SABRE reaction mixture on cells could be due to the presence of the catalyst and not the substrate (Supporting Information Figure S5). In contrast, the cells treated with various bolus volumes of [IrCl(COD)(IMes)], prepared by homogeneous mixing of the precipitate, showed loss of cell viability at higher volumes and short time points (Supporting Information Figure S6). Together these data indicate that the SABRE reaction mixture induces a decrease in cell viability at higher volumes, and this is likely due to the presence of activated catalyst. We therefore conclude that in order to achieve biocompatibility, the amount of activated catalyst must be either decrease or eliminated.

### Method of catalyst deactivation and removal

**Figure 5:**
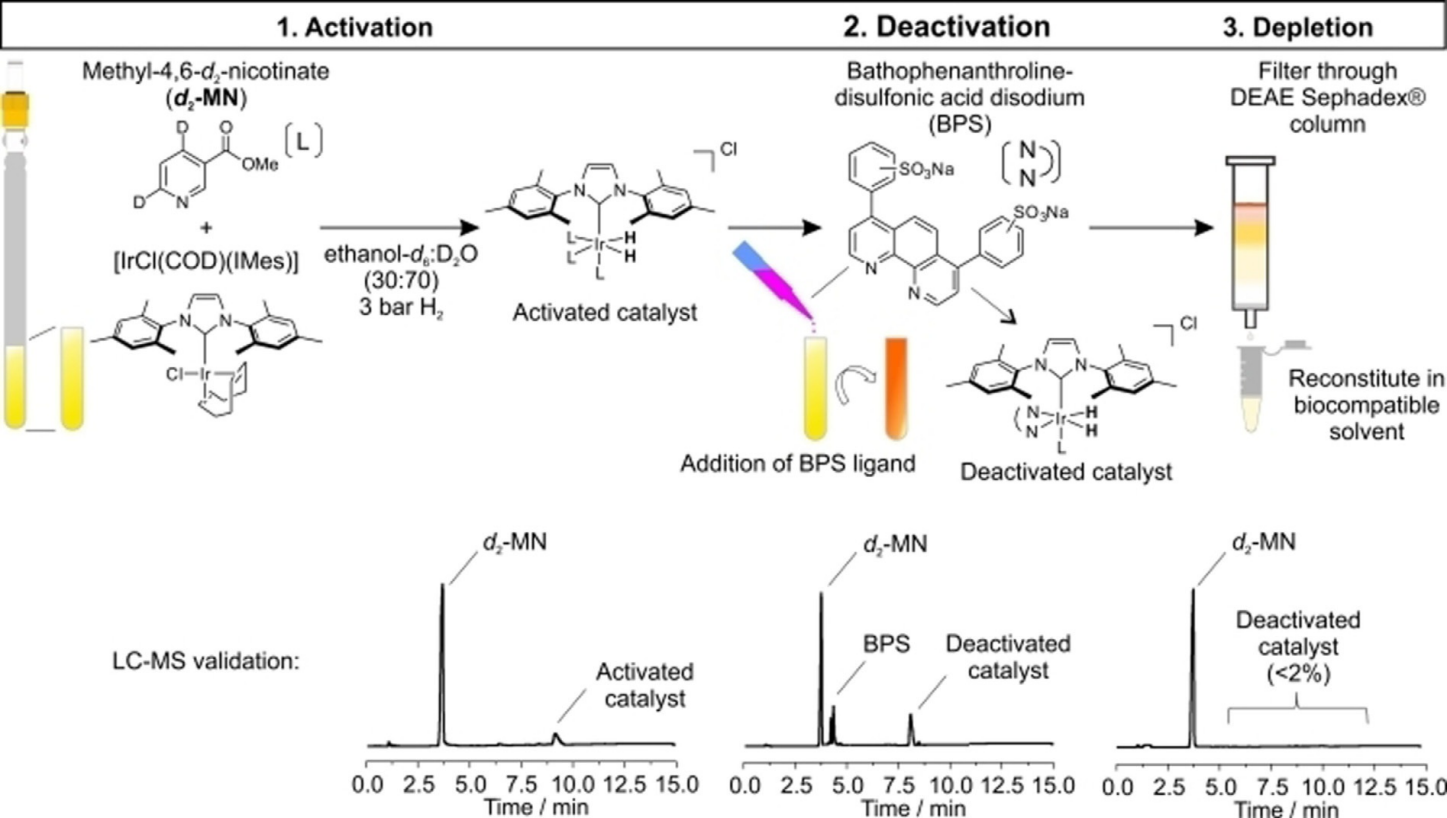
Schematic presentation of the catalyst deactivation procedure: 1. Activation: The active catalyst is formed through reaction of [IrCl(COD)(IMes)], ***d***_**2**_ **‐MN**, and H_2_ in [D_6_]ethanol/D_2_O (30:70) solution. 2. Deactivation: Addition of BPS leads to immediate formation of the deactivated catalyst [Ir(IMes)(BPS)(***d***_**2**_ **‐MN**)(H)_2_]Cl as confirmed by ^1^H NMR spectroscopy and LC–MS. 3. Depletion: Ion‐exchange chromatography on DEAE‐Sephadex® leads to <2 % catalyst contamination.

We have thus developed a protocol to remove the SABRE catalyst from solution. The addition of a chelating ligand to the SABRE reaction prevents reversible exchange of the substrate, deactivating the SABRE process without affecting the polarization levels whilst extending *T*_1_ relaxation times.^15^ We postulated that the addition of bathophenanthrolinedisulfonic acid disodium salt (BPS) would see it irreversibly bind to the iridium center whilst giving an opportunity to remove the resultant species via ion‐exchange chromatography. This procedure is shown schematically in Figure 5. First, we prepared the activated SABRE reaction mixture in [D_6_]ethanol/D_2_O solution prior to the addition of a solution of 2.0 equiv of BPS in D_2_O. Following the reaction by ^1^H NMR spectroscopy reveals the immediate formation of a new hydride species at *δ*−19.6 ppm which we attribute to [Ir(IMes)(BPS)(***d***_**2**_ **‐MN**)(H)_2_]Cl and confirmed by LC–MS (Supporting Information Figure S7). After filtration through DEAE‐Sephadex® with D_2_O as eluent, less than 2 % of the catalyst remains in solution with high mass recovery of ***d***_**2**_ **‐MN**, which can be delivered in the biocompatible [D_6_]ethanol/D_2_O solvent mixtures. This protocol is therefore efficient at removing the SABRE catalyst from solution. Importantly, the hyperpolarized SABRE signal is still visible after the deactivation and depletion steps. The total signal gains were 74±21‐fold which represents a 30 % decrease in signal relative to the standard SABRE sample in [D_6_]ethanol/D_2_O (30:70). ^1^H NMR spectra are shown in the Supporting Information (Figure S7). As the deactivation and depletion process takes a minimum of 12 seconds longer than a standard sample measurement, we attribute the loss to relaxation effects.

### Cytotoxicity assessment of catalyst depleted SABRE mixture

**Figure 6:**
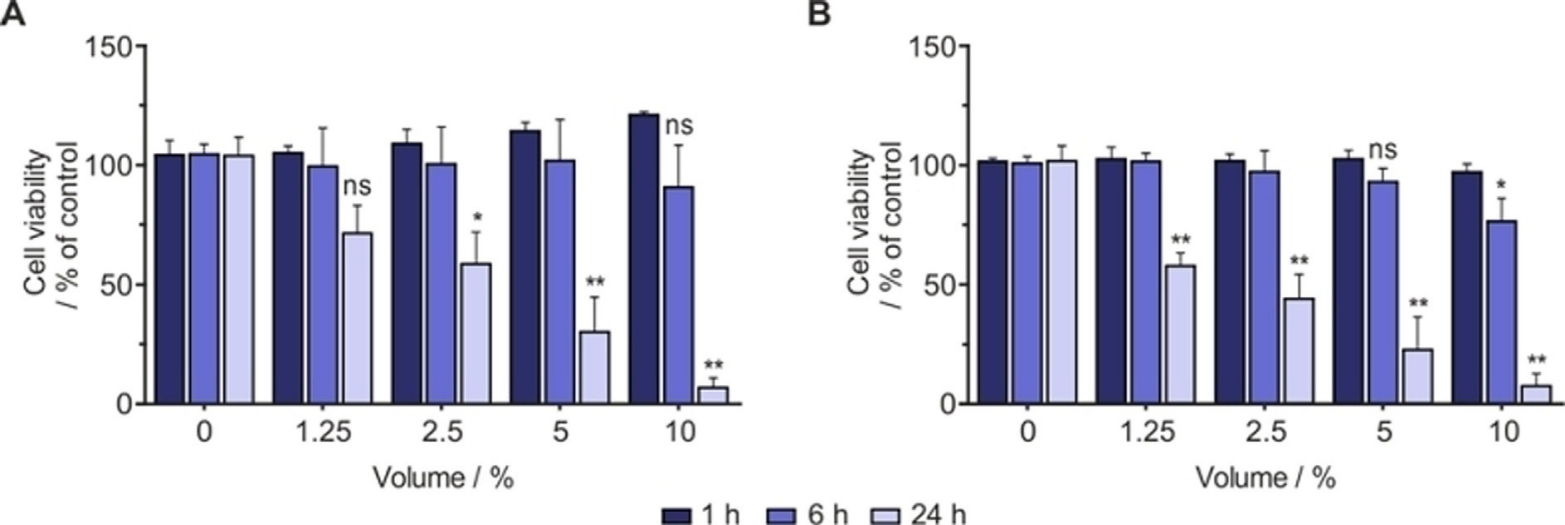
Achieving biocompatible SABRE by deactivating the catalyst: MTT viability assay showing A) A549 and B) MCF7 cells treated with various volumes of catalyst‐depleted SABRE reaction mixture for 1, 6, and 24 h. Data are the mean+SD from three independent experiments (*n*=3). *P<0.05, **P<0.005, ns: not significant vs. untreated control (100 % viable); one‐way ANOVA.

The cytotoxicity of the catalyst‐depleted samples on cells was then evaluated by taking different volumes (1.25, 2.5, 5, and 10 %) of the reconstituted mixture in [D_6_]ethanol/D_2_0 (30:70) and by following the treatment conditions in the same manner as performed with the non‐quenched SABRE reaction mixture. Pleasingly, 10 % of the bolus containing the catalyst‐depleted SABRE reaction mixture did not alter the viability of A549 cells for up to 6 h of treatment (Figure 6 A). Similarly, MCF7 cells showed no changes in cell viability when treated with higher volumes (10 %) of the catalyst‐depleted SABRE mixture for up to 1 h and for up to 6 h with lower volumes (≤5 %, Figure 6 B). However, longer treatments (24 h) showed significant decrease at both lower and higher volumes in both cell lines (Figure 6 A,B). It is noted that when extrapolating to an in vivo model, it is unlikely to show the same long‐term toxicity due to higher metabolic activity and detoxification mechanisms. Together these data indicate that deactivation and removal of the catalyst could overcome the adverse effect of the SABRE reaction mixture when treating live cells under the conditions used here.

### Achieving biocompatible SABRE by biphasic catalysis

**Figure 7:**
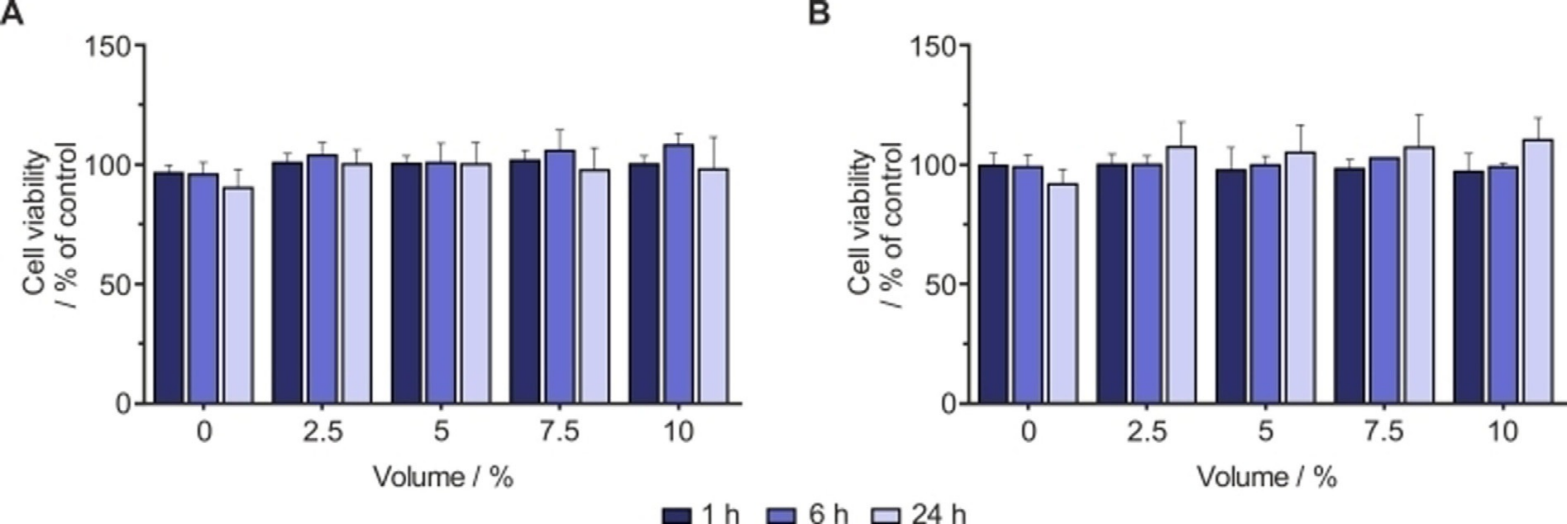
Evaluating the biocompatibility of biphasic SABRE reaction mixture: MTT viability assay showing A) A549 and B) MCF7 cells treated with various volumes (0, 2.5, 5, 7.5, and 10 %) of the bolus from the aqueous fraction of a SABRE reaction in biphasic solvents for 1, 6, and 24 h. Data are the mean+SD from three independent experiments (*n*=3).

We hypothesized that using the recently reported biphasic approach to SABRE catalysis could be a more rapid and facile way to deplete the solution of the iridium catalyst.^16^ In this method the SABRE catalyst is located in a chloroform or dichloromethane phase and minimally in the aqueous phase. Conversely, the hyperpolarized substrate is distributed between the two. For toxicity assessment on cells, the aqueous phase was isolated, and various bolus volumes (2.5, 5.0, 7.5, and 10 %) were added to the cell culture medium. We performed the appropriate viability assay at different time points as illustrated in the previous sections. Treatment with the phase‐separated SABRE mixture did not decrease the viability of either A549 or MCF7 cells at any of the time points tested (Figure 7). The minimal cytotoxic effect observed here is similar to the effect seen when treated with the substrate alone (Supporting Information Figure S9). This result indicates that the cytotoxicity associated with the SABRE reaction mixture is negated by this method. While we are able to produce a biocompatible bolus by this phase‐separation method, the polarization level achieved by the biphasic catalysis is approximately 2000‐fold for the same substrate (***d***_**2**_ **‐MN**), (Supporting Information Figure S10). Nevertheless, we conclude that polarization in the biphasic mixture is higher than that observed in an [D_6_]ethanol/D_2_O solution under 3 bar *p*‐H_2_ and at 298 K.

## Conclusions

In summary, we have shown through in vitro cytotoxicity studies that it is possible to create a biocompatible SABRE bolus for future in vivo detection. This is an important step toward preclinical/clinical disease‐state diagnosis using SABRE technology. We have exemplified our toxicity study for methyl‐4,6‐*d*_2_‐nicotinate (***d***_**2**_ **‐MN**), which shows strong hyperpolarization levels and long magnetic lifetimes.^7^ By determination of the IC_50_ values across a number of human cancer cell lines we have shown that deuteration of the substrate does not show significant change in toxicology relative to its protio analogue. This will provide important information for the future development of SABRE contrast agents. We will also determine the effects of other common isotopic labeling strategies (e.g., ^13^C and ^15^N) as we further expand our substrate profile. For example, we are interested in the tuberculosis drug isoniazid^17^ and molecules that can sustain long‐lived singlet states.4f, 18 The solvent composition was shown to have a substantial effect on cell survival rate in an in vitro assay; 100 % [D_6_]ethanol produced adverse effects on the cells over time periods of up to 6 h. We overcame this by dilution with D_2_O and found that a [D_6_]ethanol/D_2_O ratio of 30:70 showed no cell death over 24 h. Again, solvent deuteration had no effect over their protio analogues.

We have shown that the iridium catalyst is the largest contributor to decreasing the viability of cells in the SABRE mixture. Therefore, we have developed a simple and robust method to remove it from solution by ion‐exchange chromatography. To achieve this we add bathophenanthrolinedisulfonic acid disodium salt (BPS) and subsequently flush the solution through DEAE‐Sephadex®. The eluent from this procedure showed minimal adverse effects on a number of cell lines for up to 6 h exposure and retained 70 % of the initial SABRE‐induced polarization. Furthermore, the recently reported biphasic approach to SABRE catalysis has also been shown to lead to bolus biocompatibility after separation of the aqueous phase.

We are currently working toward the development of an automated delivery method that includes all of the proposed deactivation, depletion, or separation methods to exclude the catalyst that can be used in a clinical setting. Additionally, as the SABRE catalyst is the main source of toxicity, we are considering synthetic strategies to decrease toxicity through further changes to the catalyst.

## Experimental Section

**Chemicals and reagents**: All chemicals were purchased from Sigma–Aldrich, Fisher, or Alfa‐Aesar. Deuterated solvents ([D_6_]ethanol, deuterium oxide (D_2_O), and chloroform‐*d* (CDCl_3_)) were purchased from Sigma. The following compounds were prepared according to published procedures: methyl‐4,6‐*d*_2_‐nicotinate^7^ and [IrCl(COD)(IMes)].^19^

**Cell culture**: Human alveolar adenocarcinoma cells (A549), breast cancer cells (MCF7), and cervical cancer cells (HeLa) were kindly provided by Prof. Christoph Borner (IMMZ, Freiburg, Germany). The human breast adenocarcinoma cell line MDA‐MB‐231 was a gift from Prof. Thomas Kaufmann (University of Bern, Switzerland). All cell lines were grown in DMEM supplemented with 10 % fetal bovine serum (FBS), penicillin (100 U mL^−1^), streptomycin (100 μg mL^−1^), and l‐glutamine (2 mm) (all from Gibco Life Technologies). The cells were maintained in a humidified atmosphere under standard conditions (37 °C, 5 % CO_2_). The media were changed at regular intervals, and upon reaching appropriate confluence (90 %) the cells were passaged after brief exposure to a trypsin/EDTA solution (Invitrogen).

**Treatment of cells**: Immediately after trypsin/EDTA treatment, viable cells were counted in a hemocytometer by Trypan blue (Sigma) exclusion. The required number of cells (normally 10^4^ per well in a 96‐well tissue culture plate (Nunc)) was seeded 24 h before treatment so that they are in exponential growth phase at the start of the experiment. Before treatment various volumes of the bolus from substrate, catalyst, or the SABRE reaction mixture were diluted to a maximum of 10 μL in the same solvent from which the compounds were originally prepared. For cell assays, throughout this study (unless otherwise indicated) we kept the final amount of the solvent at 10 % in the total volume of the cell growth medium (i.e., 10 μL in 100 μL).

**MTT assay**: The MTT (3‐(4,5‐dimethyl‐2‐thiazolyl)‐2,5‐diphenyl‐2*H*‐tetrazolium bromide) assay is a commonly used colorimetric method to determine in vitro cytotoxicity of the given compound by means of a functional mitochondrial dehydrogenase activity in living cells.^12^ In this method, MTT tetrazolium is reduced to insoluble formazan crystals and it is directly proportional to the number living cells. Therefore, this assay represents a measure for cell viability as well. For this assay, cells (normally 10^4^ cells per well in a 96‐well plate) were taken in triplicates and treated with a range of compounds (see above) or kept as cell viability control with no compound treatment (untreated) in cell growth media. After incubation at desired time points the cell growth media was replaced with fresh media (100 μL) and incubated with 10 μL of MTT (5 mg mL^−1^, dissolved in cell growth media and filter sterilized (0.22 μm)) for 4 h in a humidified atmosphere at 37 °C. The medium with MTT was then carefully aspirated, and the formed formazan crystals were solubilized in 100 μL of dimethyl sulfoxide (DMSO). The absorbance of the colored (purple) solution was then measured at 570 nm using a microplate reader (MultiskanGO, ThermoFisher). The absorbance values (averaged out of triplicates) were blanked against DMSO, and the absorbance of cells exposed to cell growth medium only (i.e., untreated) was taken as 100 % viable (i.e., control). The cell viability of the compound‐treated samples were then calculated by normalizing to the untreated control sample and are normally expressed as percent of control. Each assay was repeated a minimum of three times for statistical analysis of the data.

**Evaluation of IC_50_**: Compounds were dissolved in cell growth media, and cells were treated with various concentrations of the compound (ranging from a maximum of 80 mm to a minimum of 1.25 mm) in 1/2 serial dilutions. Cell viability was analyzed by MTT assay after 48 h treatment. IC_50_ values of **MN** and ***d***_**2**_ **‐MN** were calculated by using GraphPad Prism software (version 5.0).

**Catalyst deactivation and removal**: A solution of [IrCl(COD)(IMes)] (5 mm) and ***d***_**2**_ **‐MN** (20 mm, 4.0 equiv) in [D_6_]ethanol/D_2_O (30:70, total volume: 3.0 mL) was degassed prior to the introduction of hydrogen at a pressure of 3 bar. After 5 min, the sample was opened to air, and a solution of bathophenanthrolinedisulfonic acid disodium salt hydrate (BPS) (10 mm, 2.0 equiv) in water (1.0 mL) was added. The resulting suspension was eluted through DEAE‐Sephadex® (2.5 g) with water (11 mL). For treatment on cells the eluent was vacuum dried and reconstituted in 30 % [D_6_]ethanol in D_2_O. Prior to treatment various volumes of the bolus were further diluted as indicated above.

**Biphasic SABRE—biphasic separation**: A solution of [IrCl(COD)(IMes)] (5 mm) and ***d***_**2**_ **‐MN** (20 mm, 4.0 equiv) in CDCl_3_ (1.5 mL) and D_2_O (containing 0.9 % NaCl) (1.5 mL) was mixed together and was degassed prior to the introduction of hydrogen at a pressure of 3 bar. After phase separation the aqueous layer was removed, and prior to treatment various volumes of the bolus were further diluted as indicated above.

**Statistical analysis**: All experiments were performed at least three times (*n*=3), and all data are presented as the mean and standard deviation (SD) of the mean. The significance of the differences between treated samples and the untreated control was assessed by one‐way analysis of variance (ANOVA) using GraphPad Prism software (version 5.0). Statistical significance was set at *P* values <0.05.

## Conflict of interest

*The authors declare no conflict of interest*.

## Supporting information

As a service to our authors and readers, this journal provides supporting information supplied by the authors. Such materials are peer reviewed and may be re‐organized for online delivery, but are not copy‐edited or typeset. Technical support issues arising from supporting information (other than missing files) should be addressed to the authors.

Supplementary
