# Supplementary material for: Achieving Biocompatible SABRE: An in vitro Cytotoxicity Study
Source: ChemMedChem. 2018 Jan 18;13(4):352–9. doi: 10.1002/cmdc.201700725 (PMC5838797; doi:10.1002/cmdc.201700725)
Supplement: Supplementary file 1 — Supplementary [file CMDC-13-352-s001.pdf]

CHEMMEDCHEM
Chemistry & Drug Discovery

Supporting Information

Achieving Biocompatible SABRE: An in vitro Cytotoxicity Study

Anand Manoharan,[a] Peter J. Rayner,[a] Wissam Iali,[a] Michael J. Burns,[a] V. Hugh Perry,[b] and Simon B. Duckett*[a]

cmdc_201700725_sm_miscellaneous_information.pdf

Table of contents

Figure S1. Effect of deuterated solvents on cell viability 2

Figure S2. Protio solvents vs. cell viability 3

Figure S3. SABRE polarisation of d_2-MN in ethanol-d_6:D_2O (30:70) 4

Figure S4. Precipitation of the catalyst 5

Figure S5. Evaluating the cytotoxicity of methyl-4,6-d_2-nicotinate prepared under SABRE conditions 6

Figure S6. Effect of the catalyst [IrCl(COD)(IMes)] alone on cell viability 7

Figure S7. ^{1}H NMR analysis of catalyst quenching and depletion 8

Figure S8. Biphasic SABRE reaction mixture 9

Figure S9. Evaluating the cytotoxicity of substrate from aqueous phase of biphasic reaction 10

Figure S10. Comparing polarisation levels of SABRE vs. Biphasic-SABRE 11

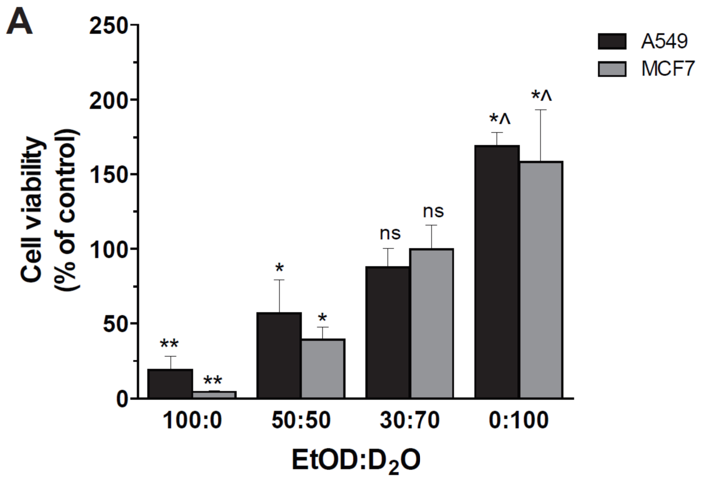

Figure S1. Effect of deuterated solvents on cell viability
Supporting figure for Figure 1. MTT assay performed on the indicated cell lines after 48 h of treatment with 10 µl of ethanol-d_6 (EtOD) 100% (100:0), 50% (50:50), 30% (30:70) and 0% (0:100) prepared in heavy water (deuterium oxide; D_2O) in a total of 100 µl of cell growth medium. The data represents mean + SD from 3 independent experiments (n=3). *P<0.05, **P<0.005 and ns - Not significant vs. the untreated control group (100% viable); one-way ANOVA. An increase in cell viability is denoted by a ‘^’.

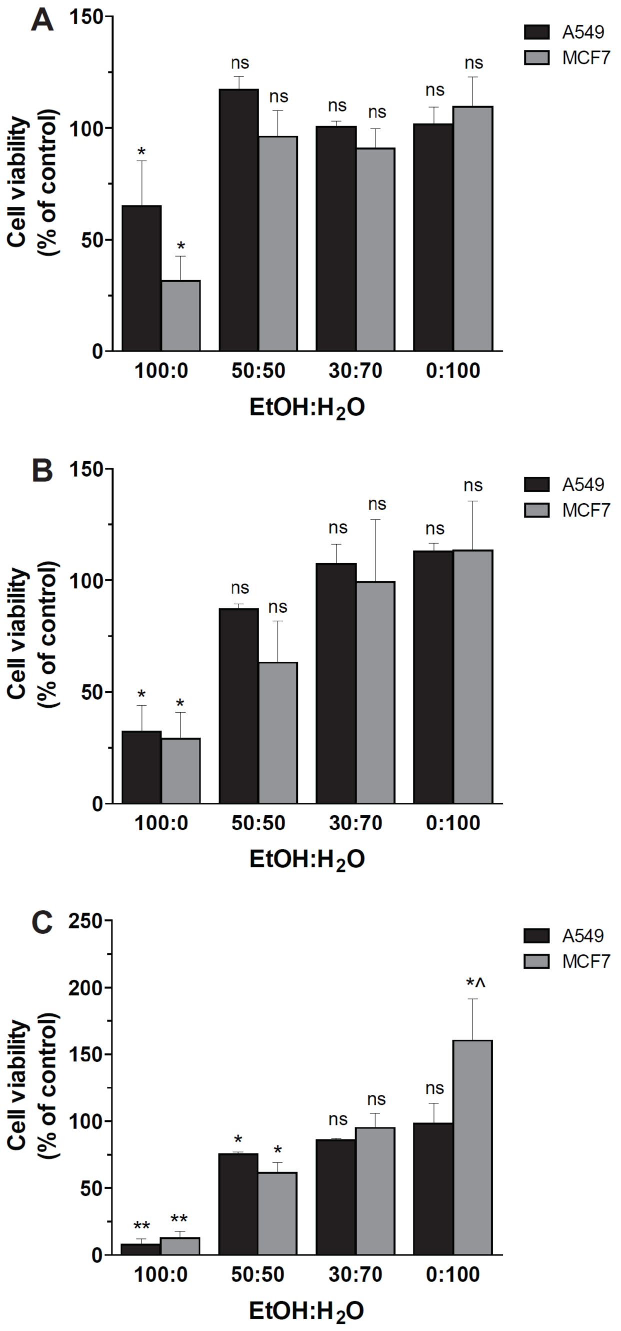

Figure S2. Protio solvents vs. cell viability

Viability data on indicated human cells treated with 10 µl of ethanol (EtOH) 100% (100:0), 50% (50:50), 30% (30:70) and 0% (0:100) prepared in water (H_2O) for (A) 6, (B) 24 and (C) 48 h in cell growth medium (100 µl). The data are presented as mean + SD and are from 3 independent experiments (n=3). *P<0.05, **P<0.005 and ns - Not significant vs. the untreated control group; one-way ANOVA. An increase in cell viability is denoted by a '^'.

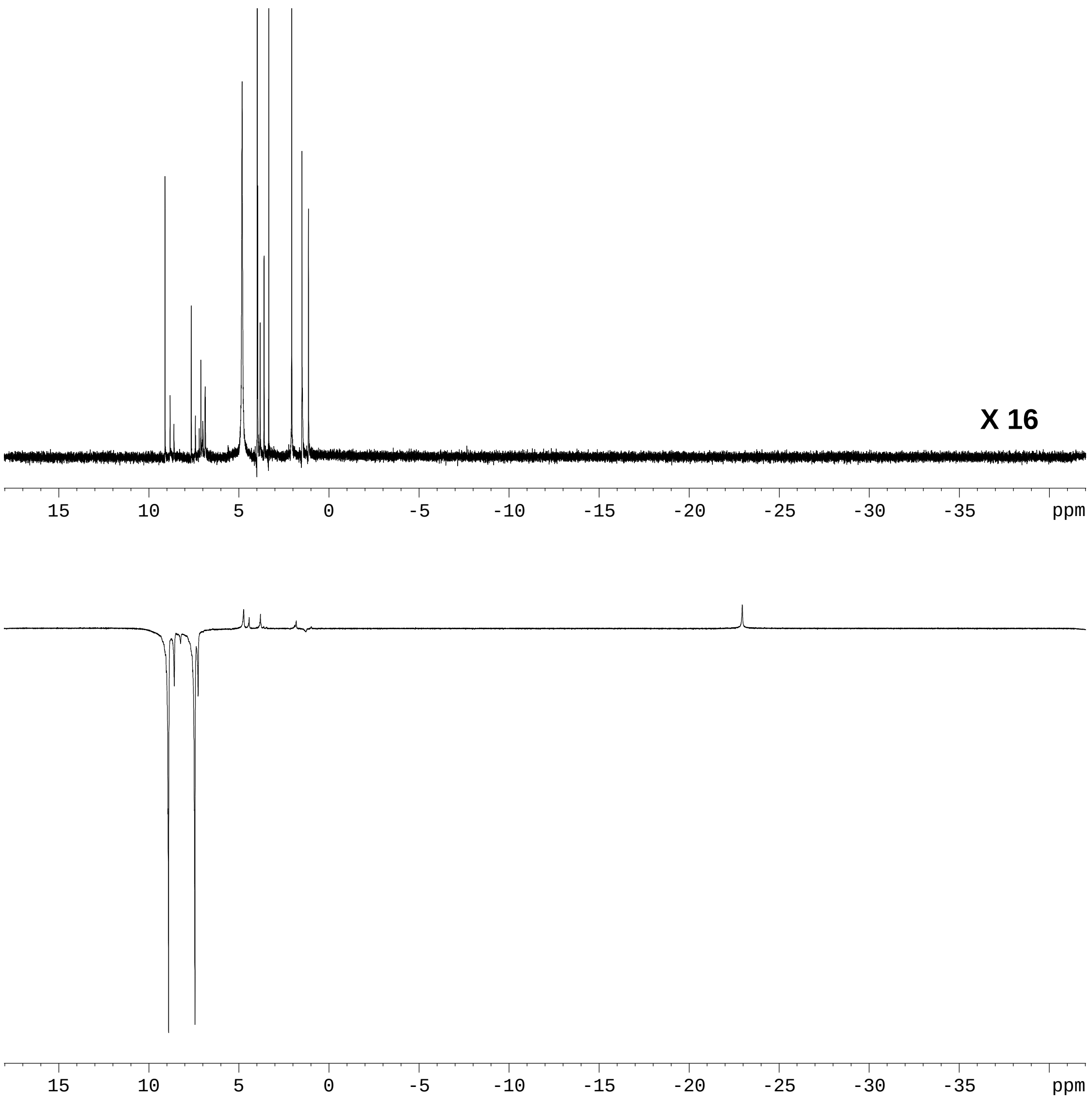

Figure S3. SABRE polarisation of *d_2*-MN in ethanol-*d_6*:D_2O (30:70)

Top - Thermally polarised ^{1}H NMR spectrum of ***d_2*-MN**, in ethanol-*d_6*:D_2O (30:70) with a x 16 vertical expansion. Bottom - Corresponding SABRE polarised ^{1}H NMR spectrum of ***d_2*-MN** after polarisation transfer at 65 G at 298 K.

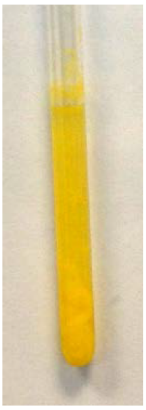

Figure S4. Precipitation of the catalyst
Picture showing NMR tube containing a precipitated solution of the catalyst [IrCl(COD)(IMes)] prepared in 30% ethanol-d_6 in D_2O after addition of H_2 at 3 bar.

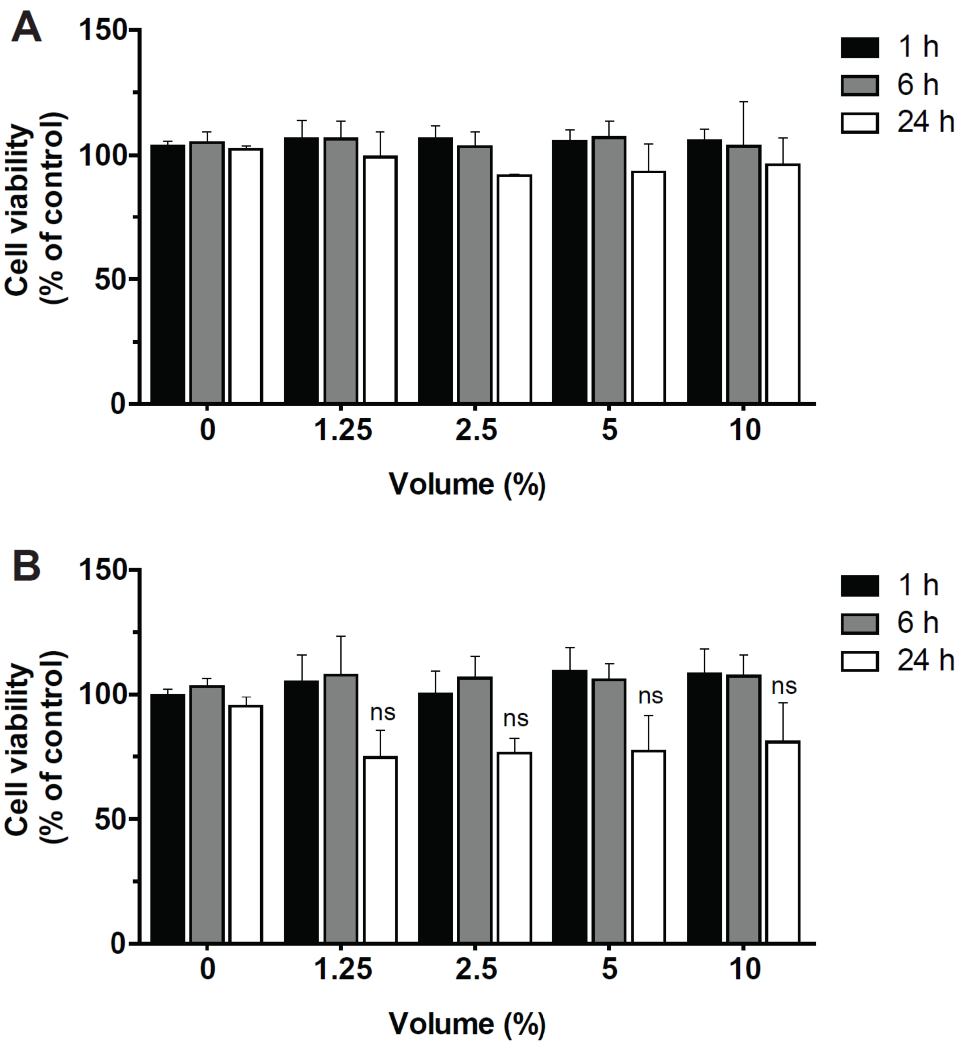

Figure S5. Evaluating the cytotoxicity of methyl-4,6-d_2-nicotinate prepared under SABRE conditions

(A) A549 and (B) MCF7 cells treated with various volumes (0, 1.25, 2.5, 5 and 10%) of the bolus containing the substrate methyl-4,6-d_2-nicotinate (***d*₂-MN**) prepared under SABRE reaction conditions but without the catalyst [IrCl(COD)(IMes)]. Prior to treatment each sample was diluted in the same solvent (30% ethanol-d_6 in D_2O) to 10 µl and then added to 100 µl of cell growth medium. Treatments were carried out for 1, 6 and 24 h and viability was determined by MTT assay. Data are the means + SD of 3 independent experiments (n=3). ns - Not significant vs. the untreated control group; one-way ANOVA.

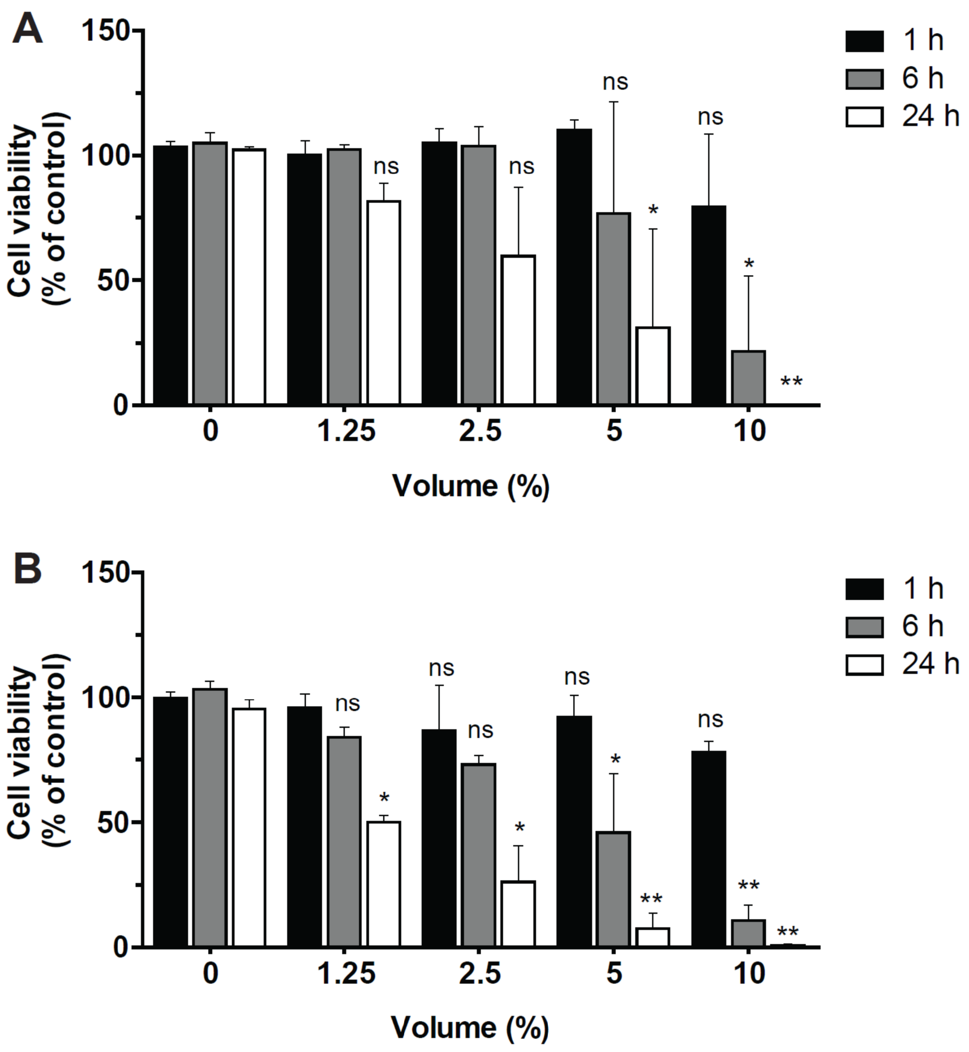

Figure S6. Effect of the catalyst [IrCl(COD)(IMes)] alone on cell viability
Cell viability data showing (A) A549 and (B) MCF7 cells treated with a solution of the catalyst [IrCl(COD)(IMes)] prepared under SABRE reaction conditions without the addition of a substrate. In the absence of a substrate or ligand [IrCl(COD)(IMes)] remained as precipitated emulsion (as shown in Figure S4). Prior to treatment different volumes (0, 1.25, 2.5, 5 and 10%) of the emulsion was taken as bolus under constant mixing and diluted further to 10 µl in the same solvent (30% ethanol-d_6 in D_2O). Cells were treated for 1, 6 and 24 h and viability was estimated by MTT assay. The data are presented as mean + SD and are from 3 independent experiments (n=3). *P<0.05, **P <0.005 and ns - Not significant vs. the untreated control group; one-way ANOVA.

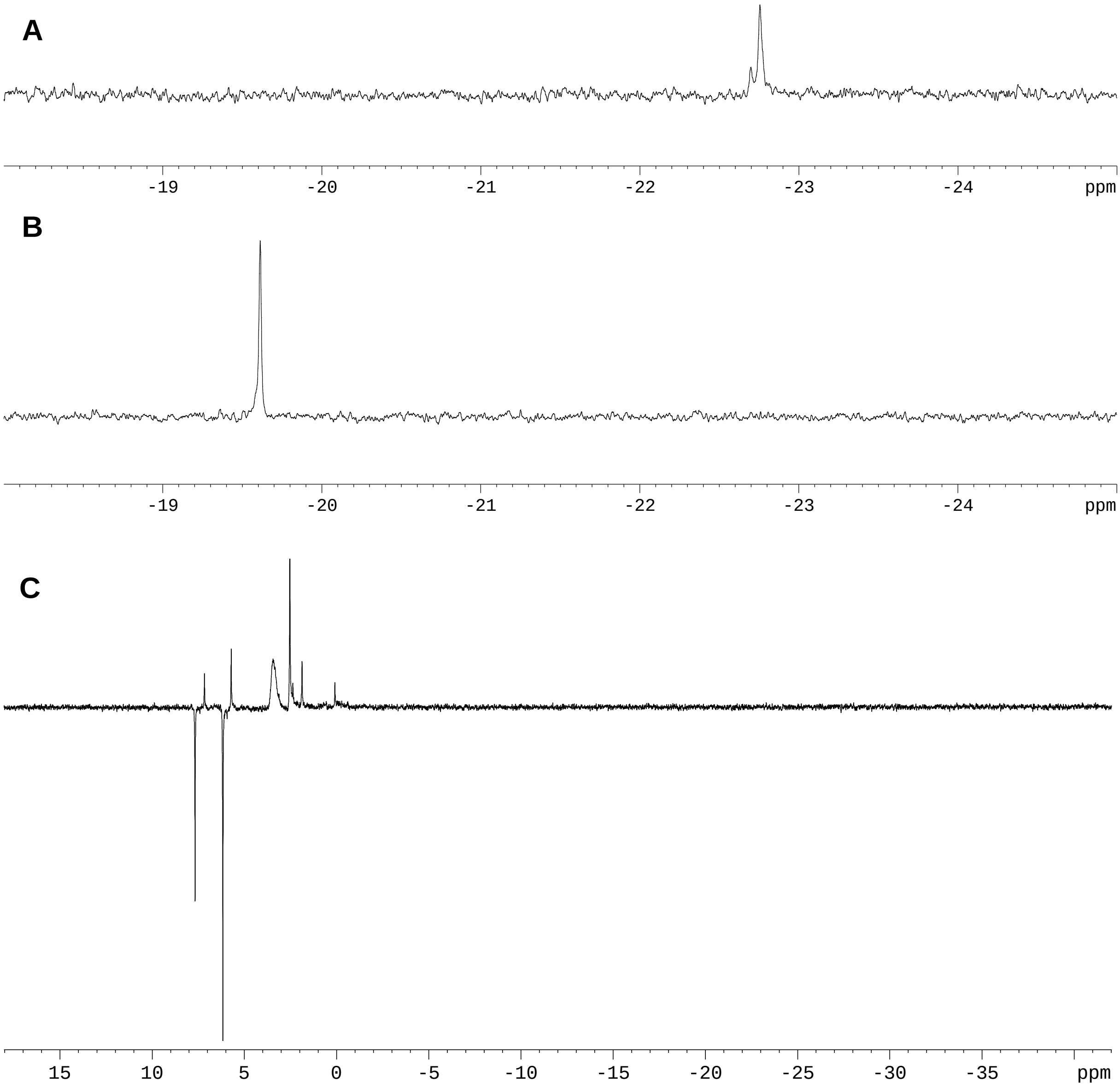

Figure S7. ^{1}H NMR analysis of catalyst quenching and depletion
Hydride regions of the ^{1}H NMR spectra of (A) [Ir(IMes)(***d*₂-MN**)$_3$(H)$_2$]Cl and (B) [Ir(IMes)(BPS)(***d*₂-MN**)(H)$_2$]Cl immediately after addition of BPS. (C) SABRE polarised ^{1}H NMR spectrum of the catalyst depleted mixture after filtration through DEAE-Sephadex.

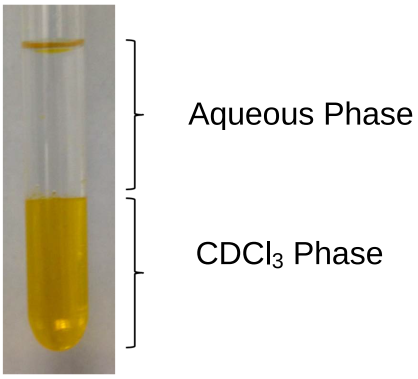

Figure S8. Biphasic SABRE reaction mixture

NMR tube containing an equal volume of chloroform-*d* ($CDCl_3$) and saline (0.9% of NaCl) in D_2O. The solution contained the catalyst [IrCl(COD)(IMes)] (5 mM) and the substrate methyl-4,6-d_2-nicotinate (***d***$\boldsymbol{_2}$**-MN**) (4 eq.;20 mM). Phase separation is clearly seen with upper aqueous and lower organic phase ($CDCl_3$). The catalyst [IrCl(COD)(IMes)] is a yellow coloured powder dissolved well in $CDCl_3$ (insoluble in D_2O) and is expected to remain in the organic phase[1]

1. W. Iali, A. M. Olaru, G. G. R. Green and S. B. Duckett, *Chemistry – A European Journal*, DOI: 10.1002/chem.201702716, DOI: 10.1002/chem.201702716.

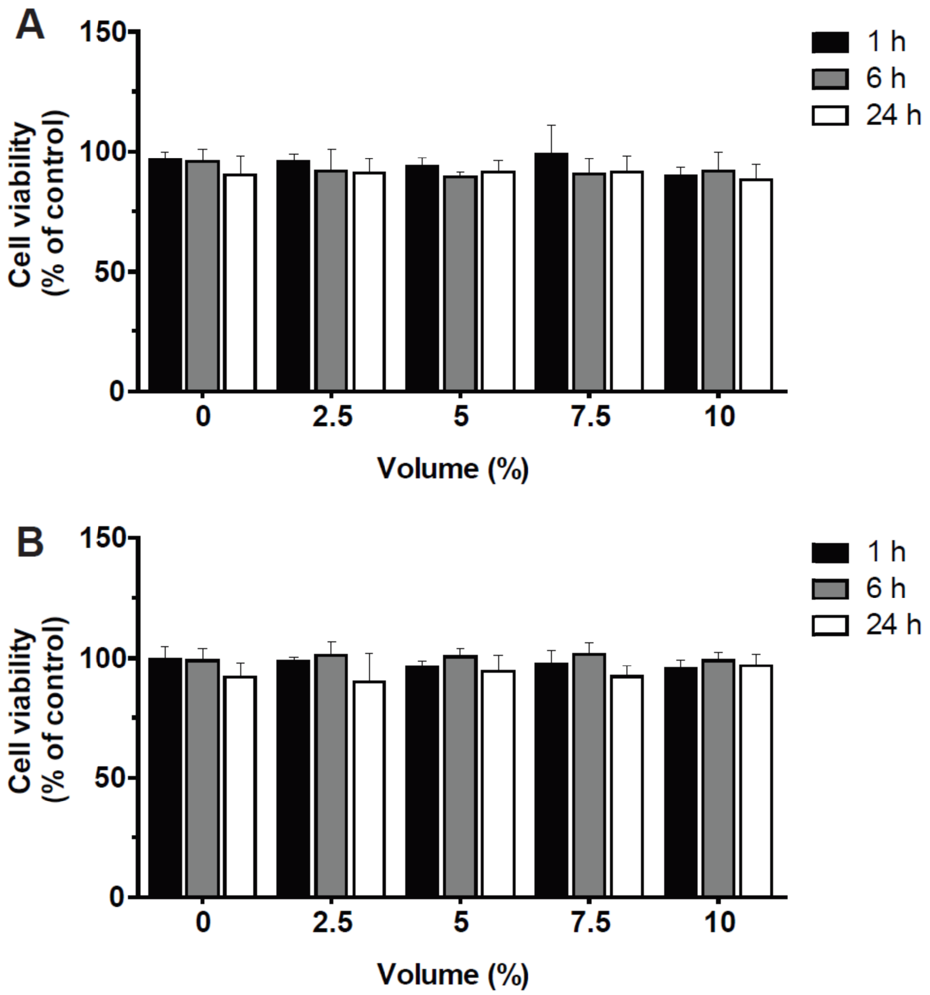

Figure S9. Evaluating the cytotoxicity of substrate from aqueous phase of biphasic reaction Cytotoxicity data showing (A) A549 and (B) MCF7 cells treated with various volumes (0, 2.5, 5, 7.5 and 10%) of the bolus from the aqueous fraction of a biphasic mixture (see Figure S8) that contained the substrate ***d*₂-MN** alone. Prior to treatment each sample was diluted further to 10 µl in the same fraction (0.9% NaCl in D_2O). Cell viability was measured by MTT assay after 1, 6 and 24 h of treatment. The data are expressed as mean + SD and are from 3 independent experiments (n=3).

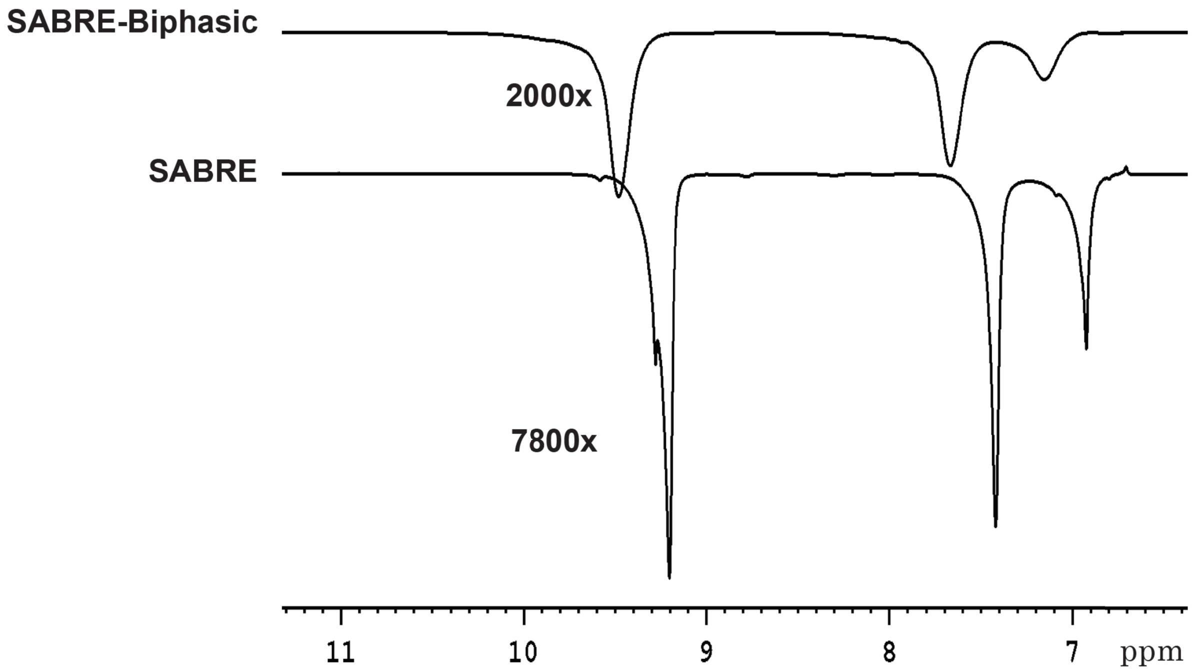

Figure S10. Comparing polarisation levels of SABRE vs. Biphasic-SABRE
^{1}H NMR spectra resulting from hyperpolarised SABRE and SABRE-biphasic analysis of the substrate methyl-4,6-d_2-nicotinate (***d*₂-MN**), prepared according to the legend of Figure S8. The signal enhancements (fold) are detailed within the figure and establish the viability of the biphasic process.
